# 5-Heptadecylresorcinol Improves Aging-Associated Hepatic Fatty Acid Oxidation Dysfunction via Regulating Adipose Sirtuin 3

**DOI:** 10.3390/nu16070978

**Published:** 2024-03-27

**Authors:** Kuiliang Zhang, Lei Jiang, Lamei Xue, Yu Wang, Yujie Sun, Mingcong Fan, Haifeng Qian, Li Wang, Yan Li

**Affiliations:** State Key Laboratory of Food Science and Technology, School of Food Science and Technology, Jiangnan University, Wuxi 214122, China; zkl931118@163.com (K.Z.); 6210113183@stu.jiangnan.edu.cn (L.J.); 6170112095@stu.jiangnan.edu.cn (L.X.); 7210112055@stu.jiangnan.edu.cn (Y.W.); 19850158201@163.com (Y.S.); fanmingcong@yeah.net (M.F.); qianhaifeng@jiangnan.edu.cn (H.Q.)

**Keywords:** adipose Sirt3, aging, 5-heptadecylresorcinol, fatty acid oxidation, liver

## Abstract

Aging-associated hepatic fatty acid (FA) oxidation dysfunction contributes to impaired adaptive thermogenesis. 5-Heptadecylresorcinol (AR-C17) is a prominent functional component of whole wheat and rye, and has been demonstrated to improve the thermogenic capacity of aged mice via the regulation of Sirt3. However, the effect of AR-C17 on aging-associated hepatic FA oxidation dysfunction remains unclear. Here, 18-month-old C57BL/6J mice were orally administered with AR-C17 at a dose of 150 mg/kg/day for 8 weeks. Systemic glucose and lipid metabolism, hepatic FA oxidation, and the lipolysis of white adipose tissues (WAT) were measured. The results showed that AR-C17 improved the hepatic FA oxidation, and especially acylcarnitine metabolism, of aged mice during cold stimulation, with the enhancement of systemic glucose and lipid metabolism. Meanwhile, AR-C17 improved the WAT lipolysis of aged mice, promoting hepatic acylcarnitine production. Furthermore, the adipose-specific Sirt3 knockout mice were used to investigate and verify the regulation mechanism of AR-C17 on aging-associated hepatic FA oxidation dysfunction. The results showed that AR-C17 failed to improve the WAT lipolysis and hepatic FA oxidation of aged mice in the absence of adipose Sirt3, indicating that AR-C17 might indirectly influence hepatic FA oxidation via regulating WAT Sirt3. Our findings suggest that AR-C17 might improve aging-associated hepatic FA oxidation dysfunction via regulating adipose Sirt3.

## 1. Introduction

Aging is characterized by a systemic physiological decline that is accompanied by transcriptional, epigenetic, and metabolic changes [1]. Recently, twelve hallmarks of aging have been proposed, including epigenetic alterations, telomere attrition, genomic instability, disabled macroautophagy, loss of proteostasis, altered intercellular communication, mitochondrial dysfunction, cellular senescence, deregulated nutrient-sensing, dysbiosis, stem cell exhaustion, and chronic inflammation [2]. These hallmarks are interrelated with each other, closely associating with cardiovascular disorders, type 2 diabetes, obesity, and cancer in elderly individuals [3]. As the largest metabolic organ, the liver is a hub for maintaining homeostasis throughout the human body [4]. The liver has a wide range of physiological functions, such as molecular biosynthesis, energy metabolism, and the removal of xenobiotics [5]. Liver aging contributes to an elevated vulnerability to acute injury and liver diseases and susceptibility to the fibrotic and inflammatory responses, which are related to aging-associated diseases such as diabetes and cardiometabolic diseases [6]. Aging-associated hepatic changes include volume loss, inflammation, reduced regeneration ability, and progressive organ dysfunction [5,7]. Liver aging results from various intrinsic and extrinsic factors, including alterations of the genome and epigenome, mitochondrial dysfunction, and impaired nutrient sensing pathways [5]. Therefore, liver aging seriously affects the metabolic health of the elderly, and multi-angle approaches are promising to counteract liver aging.

The liver is regarded as a hub in adaptive thermogenesis [8]. Upon the activation of adaptive thermogenesis, cold stimulates the lipolysis of white adipose tissue (WAT) to release free fatty acid (FFA) that activates acylcarnitine production and provides the substrate for acylcarnitine synthesis in the liver [9]. Acylcarnitines are intermediate products of mitochondrial FA oxidation. During cold stimulation, circulating FFA derived from WAT lipolysis enters into hepatocytes [9]. Once in the cytoplasm, FA is activated by acyl-CoA synthetase and conjugated with carnitine to convert to acylcarnitine with carnitine palmitoyltransferase 1a (CPT1a) [10]. Acylcarnitine allows the transportation of FA across the inner mitochondrial membrane through carnitine-acylcarnitine translocase (CACT) [11]. In the mitochondrial matrix, acylcarnitines are transformed to carnitine and fatty acyl-CoAs by carnitine palmitoyltransferase 2 (CPT2) for subsequent β-oxidation [9]. During cold stimulation, acylcarnitines synthesized in the liver are released to the circulation, through which the acylcarnitines are transported to brown adipose tissue (BAT) for thermogenesis [9]. Therefore, cold stimulation mobilizes FAs from WAT, resulting in the enhancement of hepatic FA oxidation and the production of acylcarnitines that are delivered to BAT for thermogenesis. The liver is a nexus for the organ crosstalk of WAT–liver–BAT and an indispensable organ for BAT thermogenesis.

The higher and frequent consumption of whole grains (WG) is associated with low risks of developing aging-related diseases and the extension of healthy life expectancy [12,13]. 5-n-Alkylresorcinols (ARs) are biomarkers of WG rye and wheat intake, existing in the outer layer of rye and wheat with various health promoting effects [14,15]. 5-Heptadecylresorcinol (AR-C17) is a major active homolog of ARs and has been demonstrated to have various health benefits, including anti-oxidant, anti-obesity, anti-inflammatory, and anti-cancer activities [16,17,18,19]. AR-C17 has been demonstrated to modulate various metabolic processes via regulating Sirtuin 3 (Sirt3) [16,20,21,22]. Sirt3 is a human Sir2 homologue which translocates into mitochondria under cellular stress [23]. Upon entering the mitochondria, Sirt3 displays deacetylation activity in a nicotinamide adenine dinucleotide (NAD)-dependent manner [24]. Sirt3 plays a crucial role in the regulation of energy metabolism and can regulate many metabolic processes, such as oxidative phosphorylation, FA oxidation, and the tricarboxylic acid cycle [25]. In our previous study, we showed that AR-C17 improved the metabolism of thermogenic fat and the thermogenic capacity of aged mice via the regulation of Sirt3 [22]. Furthermore, we also revealed that AR-C17 improved the BAT thermogenesis of aged mice via enhancing the acylcarnitine metabolism of BAT [26]. Since acylcarnitine production in the liver is closely associated with BAT thermogenesis, we wonder whether AR-C17 could regulate the acylcarnitine production of the liver in aged mice during cold stimulation. AR-C17 can be detected in plasma and adipose tissue after the long-term dietary intake of whole grain [27]. Therefore, whether AR-C17 could enter into the liver to directly regulate hepatic FA oxidation remains unclear. Meanwhile, providing that AR-C17 could not directly regulate hepatic FA oxidation, we speculated that AR-C17 might influence hepatic FA oxidation through the organ crosstalk of WAT–liver–BAT.

In the present study, we found that AR-C17 improved the glucose and lipid metabolism of aged mice, with the recovery of the cold sensitive phenotype. Furthermore, AR-C17 might improve the hepatic FA oxidation, especially acylcarnitine metabolism, of aged mice during cold stimulation. Meanwhile, AR-C17 improved the WAT lipolysis of aged mice, which promotes hepatic acylcarnitine production. To investigate whether AR-C17 directly regulates hepatic FA oxidation independent of adipose, we established the adipose-specific Sirt3 knockout (Sirt3 AKO) mice, removing the regulatory target of AR-C17 in adipose. Our results showed that AR-C17 failed to improve the WAT lipolysis and hepatic FA oxidation of aged mice in the absence of adipose Sirt3. Our findings suggest that AR-C17 improves aging-associated hepatic FA oxidation dysfunction by regulating adipose Sirt3, contributing to the enhancement of thermogenesis in aged mice.

## 2. Materials and Methods

### 2.1. Materials and Reagents

AR-C17 (purity ≥ 98%) was purchased from Macklin (Shanghai, China). TRIzol Reagent was purchased from Thermo Fisher Scientific (San Diego, CA, USA). Hifair^®^ III 1st Strand cDNA Synthesis SuperMix and Hieff^®^ qPCR SYBR Green Master Mix were purchased from Yeasen Biotechnology Co., Ltd. (Shanghai, China). The antibody against PPARα (sc-398394) was obtained from Santa Cruz Biotechnology. Antibodies against CPT1a (15184-1-AP), CACT (19363-1-AP), and CPT2 (26555-1-AP) were obtained from Proteintech (Wuhan, China). Antibodies against ATGL (2439), phospho-HSL (4137), HSL (18381), Sirt3 (5490), and β-actin (3700) were obtained from Cell Signaling Technology (Danvers, MA, USA). RIPA lysis buffer and BCA Protein Quantification Kit were obtained from Beyotime (Shanghai, China). Total cholesterol (TC) and triglyceride (TG) assay kits were obtained from Nanjing Jiancheng Bioengineering Institute (Nanjing, China). FFA content assay kit was obtained from Solarbio (Beijing, China). All other reagents were from Sigma Chemical Co. (Beijing, China) unless stated otherwise.

### 2.2. Animal and Experimental Design

All animal experiments were performed according to procedures approved by the Laboratory Animal Ethics Committee of Jiangnan University (JN.No20220515c0021201[157], JN.No20220615c0480930[246]) and complied with guidelines and regulations of the Guide for the Care and Use of Laboratory Animals. All mice were kept in a specific pathogen-free animal facility with a 12 h dark–light cycle at a constant temperature of 23 ± 2 °C and relative humidity of 55 ± 5%. All mice were given free access to a normal chow diet (AIN-93G) and water. Young C57BL/6J male mice aged 12 weeks and aging male mice aged 18 months were purchased from Charles River. The mice were divided into three groups: the young mouse group, the aged mouse group, and the aged mice administered with AR-C17 group. Sirt3 AKO mice were generated using the Cre-lox system by crossing Sirt3^fl/fl^ mice (Strain#031201) with adiponectin-Cre^+/−^ mice (Strain#028020), which were all purchased from the Jackson Laboratory. The offspring Sirt3^fl/fl^ mice served as the littermate control, labelled Floxed mice. The experiment began when the bred mice were 18 months old. The mice were divided into three groups: the Floxed mouse group, the Sirt3 AKO mouse group, and the Sirt3 AKO mice administered with AR-C17 group. AR-C17 was dissolved in 0.5% CMC-Na (Macklin, Shanghai, China) aqueous solution, and the control groups received the same volume of CMC-Na aqueous solution. AR-C17 was orally administered to mice at the dose of 150 mg/kg/day for 8 weeks [22]. At the end of the treatment, the mice were received cold exposure (4 °C) for 48 h, and rectal temperature was measured using a rectal thermometer (RWD Life Science, Shenzhen, China) during cold exposure. All the mice were euthanized with CO_2_, and the plasma, serum, liver, inguinal WAT (iWAT), and epididymal WAT (eWAT) were immediately harvested and stored at −80 °C for further analyses.

### 2.3. Glucose Tolerance Test (GTT) and Insulin Tolerance Test (ITT)

GTT was carried out on mice by intraperitoneal injection with 1 g/kg body weight of D-glucose after fasting for 16 h. For ITT, mice were injected intraperitoneally with regular human insulin (Novolin^®^ 30R Penfill^®^, Novo Nordisk, Bagsværd, Denmark) at a dose of 0.75 U/kg body weight. Blood glucose levels were measured from the tail vein at 0, 15, 30, 60, and 120 min after injection using a blood glucose meter (ACCU-CHEK Instant, Roche, Basel, Switzerland).

### 2.4. Biochemical Analysis of Serum

The total cholesterol (TC) and triglyceride (TG) levels in serum were measured according to the manufacturer’s protocols (Nanjing Jiancheng Bioengineering Institute, Nanjing, China). Briefly, each serum sample was added to the well of 96-well plates and then mixed with the enzyme reagent provided in the assay kit. After incubation at 37 °C for 10 min, the absorbance of each well was measured using a Microplate reader (Thermo Fisher Scientific, Waltham, MA, USA). The serum FFA was measured according to the manufacturer’s protocols (Solarbio, Beijing, China). Briefly, each serum sample was treated with n-Heptane/absolute methanol/chloroform (24:1:25) and centrifugated. Then, the supernatant was used to determine the FFA levels according to the manufacturer’s protocols.

### 2.5. Histological Analysis

The liver tissues were soaked in 4% paraformaldehyde for 24 h and then embedded in paraffin. The embedded paraffin blocks were cut into 5 μm sections using an automatic constant temperature freezing microtome (Leica CM1950, Leica, Berlin, Germany). The sections were stained with Hematoxylin and Eosin (H&E) after deparaffinization and rehydration. Finally, the stained slides were sealed with resinene and observed by inverted light microscopy (ZEISS Axio Vert A1, Leica, Berlin, Germany).

### 2.6. RNA Isolation and qRT-PCR Analysis

Total RNA was extracted from tissues and cells using Trizol reagent. All RNA was quantified using a One Drop spectrophotometer. cDNAs were synthesized using Hifair^®^ III 1st Strand cDNA Synthesis SuperMix. qRT-PCR was performed using Hieff^®^ qPCR SYBR Green Master Mix on a QuantStudio 3 RT-PCR system (Applied Biosystem, Waltham, MA, USA). As an internal control, β-actin was used to normalize the data to determine the relative expressions of the target genes by using the 2^−ΔΔCt^ method. Primer sequences used for qRT-PCR are shown in Table S1.

### 2.7. Western Blotting

Samples were homogenized and extracted in RIPA lysis buffer with a phosphatase and protease inhibitor cocktail, following 10 min boiling and centrifugation to collect the supernatant for subsequent analyses. The protein concentration was determined using a BCA Protein Quantification Kit. Samples containing equal amounts of protein were loaded and separated on 10% SDS-PAGE and subsequently transferred to polyvinylidene difluoride membranes. Membranes were incubated with primary antibodies overnight at 4 °C. After washing three times with TBST, the membranes were incubated with Peroxidase-AffiniPure Goat Anti-Rabbit/Mouse IgG (Jackson ImmunoResearch, West Grove, PA, USA) at a dilution of 1:1000 for 1 h. Then, the membranes were washed another three times with TBST. Finally, the blots were visualized using an X-ray film processor (Delight, Suzhou, China). Protein band intensities were quantified in Image J (version 1.6, NIH, Bethesda, MD, USA) software.

### 2.8. Acylcarnitine Analysis

The pretreatment of samples for acylcarnitine analysis was conducted as previously published, with some modifications [28]. Briefly, 50 mg frozen tissue of mice were separately placed in the centrifuge tubes for extraction. Individual samples were added to 500 μL cold methanol and homogenized at 4 °C, and then the mixtures were sonicated for 15 min in ice water bath. Metabolite extracts were isolated by centrifugation at 12,000× *g* for 15 min at 4 °C, and the supernatants were separated and completely dried using lyophilization. The dried samples were re-dissolved into 20% methanol for targeted metabolomics analysis. Liquid chromatography–mass spectrometry-based targeted metabolite analysis was performed on a LC-QTRAP 5500^+^-MS/MS (Sciex, Concord, Vaughan, ON, USA). LC separations were carried out on a Kinetex C18 column (100 × 2.1 mm, particle size 2.6 μm, Phenomenex, Torrance, CA, USA) on reverse phase mode for 24 min. Column temperature and flow rate were set to 40 °C and 0.30 mL/min, respectively. The binary gradient system consisted of 0.1% formic acid in water (solvent A) and acetonitrile (solvent B). The linear gradient used for elution and equilibration of the initial gradient for subsequent runs was 5% B from 0 to 2 min, 5–45% B from 2 to 5 min, 45–100% B from 5 to 15 min, 100% B from 15 to 19 min, 100–5% B from 19 to 20 min, and 5% B from 20 to 24 min. The unlabeled and labeled Carnitine Standards Set B (Cambridge Isotope Laboratories, Tewksbury, MA, USA) were used for the standard.

### 2.9. Statistical Analysis

All values were presented as mean ± standard error mean (SEM). One-way ANOVA with post hoc analyses were performed for the assessment of the difference between three groups. Statistical analysis was performed using GraphPad Prism 8.0 software (GraphPad Software, La Jolla, CA, USA). The significance was presented as * for *p* < 0.05, ** for *p* < 0.01, and *** for *p* < 0.001. All experiments were performed at least three times, and representative data were shown.

## 3. Results

### 3.1. AR-C17 Improved Glucose and Lipid Metabolism during Aging

**Figure 1:**
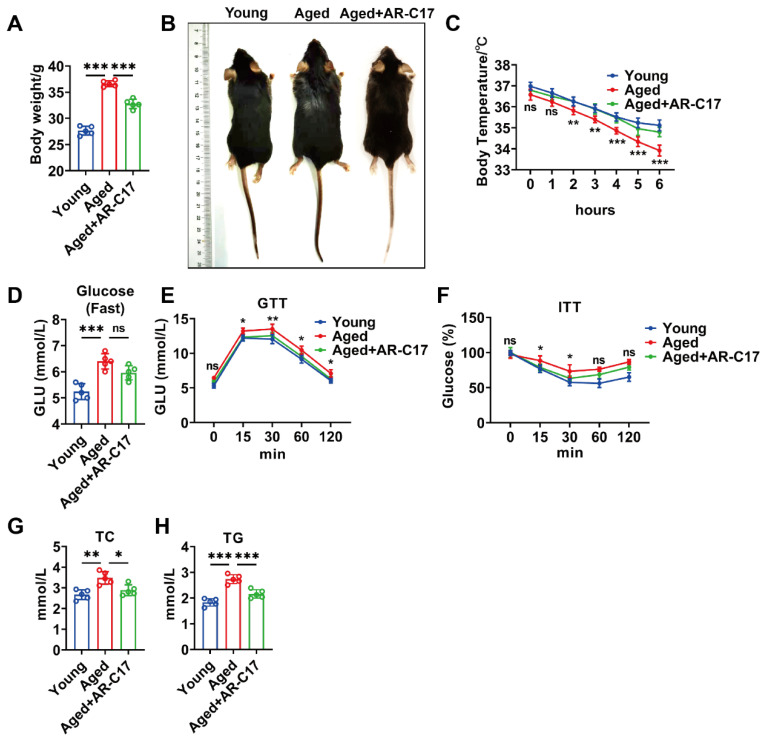
AR-C17 improved glucose and lipid metabolism during aging. (**A**) Body weight of the young mice, the aged mice, and the aged mice administered with AR-C17 (*n* = 5). (**B**) Representative photograph of the young mice, the aged mice, and the aged mice administered with AR-C17. (**C**) Core body temperature of the young mice, the aged mice, and the aged mice administered with AR-C17 during cold exposure (*n* = 5). (**D**–**F**) Fasting blood glucose, GTT, and ITT of the young mice, the aged mice, and the aged mice administered with AR-C17 at room temperature (*n* = 5). (**G**,**H**) Serum TC and serum TG of the young mice, the aged mice, and the aged mice administered with AR-C17 after cold exposure (*n* = 5). Data are presented as the mean ± SEM and n indicates the number of biologically independent experiments. Statistical significance was determined using one-way ANOVA with post hoc analyses. * *p* < 0.05; ** *p* < 0.01; *** *p* < 0.001; ns, not statistically significant.

To investigate the effect of AR-C17 on systemic glucose and lipid metabolism during aging, aged mice received AR-C17 administration for 8 weeks. The results showed that aged mice had a higher body weight and larger body size compared with young mice, and AR-C17 decreased the body weight and body size of aged mice (Figure 1A,B). During cold stimulation, aged mice displayed a lower body temperature than young mice, and AR-C17 increased the body temperature of aged mice (Figure 1C), indicating that AR-C17 reversed the cold-sensitive phenotype of aged mice. Furthermore, AR-C17 improved the glucose tolerance and insulin sensitivity of aged mice at room temperature, without the significant changes in fasting blood glucose levels (Figure 1D–F). Meanwhile, the TC and TG levels in the serum of aged mice were higher than in young mice, and AR-C17 reduced the high levels of TC and TG in the serum of aged mice after cold exposure (Figure 1G,H). The findings suggested that AR-C17 improved the glucose metabolism of aged mice at room temperature and the lipid metabolism of aged mice after cold exposure.

### 3.2. AR-C17 Improved Hepatic Fatty Acid Oxidation during Aging

**Figure 2:**
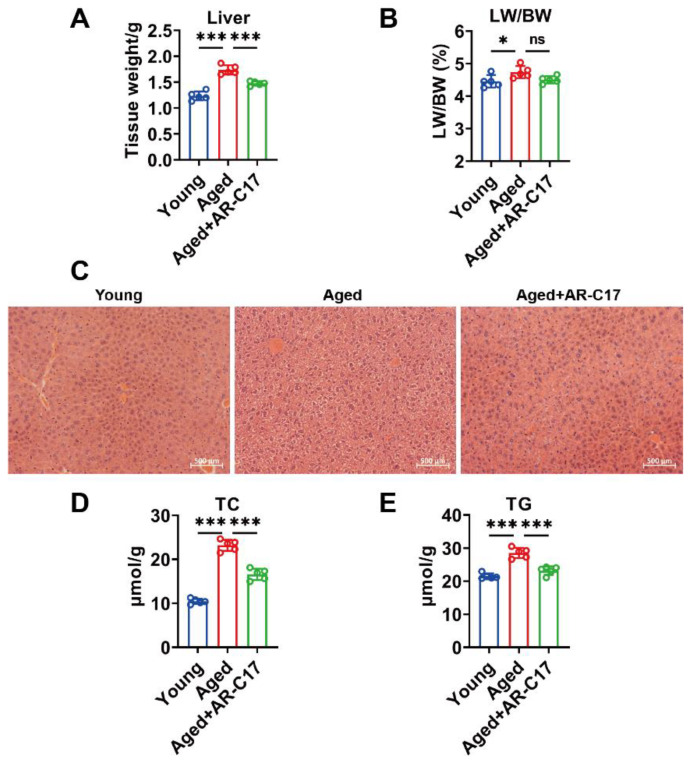
AR-C17 alleviated lipid accumulation in the liver during aging after cold exposure. (**A**) The liver weight of the young mice, the aged mice, and the aged mice administered with AR-C17 after cold exposure (*n* = 5). (**B**) The ratio of liver weight to body weight (LW/BW) of the young mice, the aged mice, and the aged mice administered with AR-C17 after cold exposure (*n* = 5). (**C**) Representative H&E staining of the liver from the young mice, the aged mice, and the aged mice administered with AR-C17 after cold exposure. (**D**,**E**) Liver TC (**D**) and TG (**E**) levels of the young mice, the aged mice, and the aged mice administered with AR-C17 after cold exposure (*n* = 5). Data are presented as the mean ± SEM and n indicates the number of biologically independent experiments. Statistical significance was determined using one-way ANOVA with post hoc analyses. * *p* < 0.05; *** *p* < 0.001; ns, not statistically significant.

**Figure 3:**
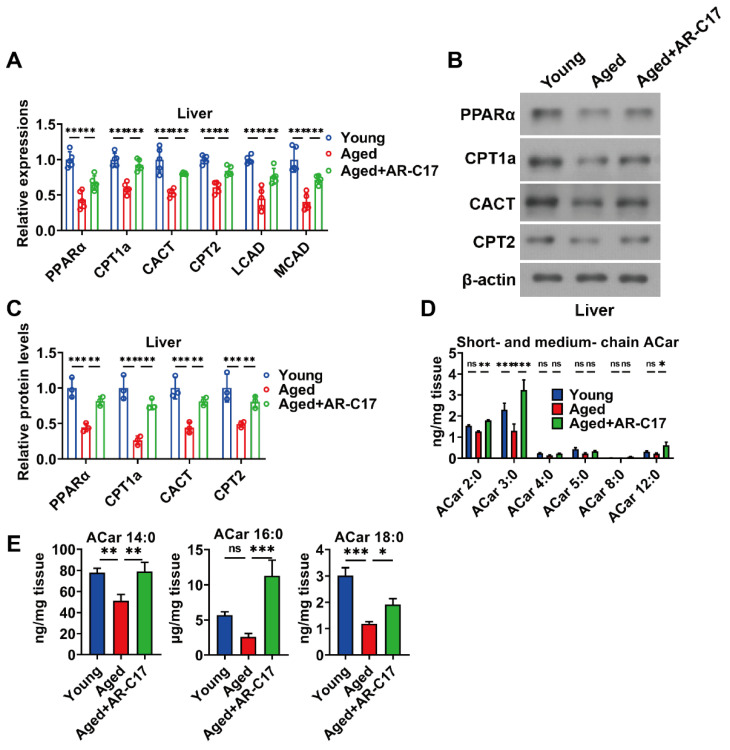
AR-C17 improved hepatic FA oxidation during aging after cold exposure. (**A**) Relative mRNA levels of PPARα, CPT1a, CACT, CPT2, LCAD, and MCAD in liver of the young mice, the aged mice, and the aged mice administered with AR-C17 after cold exposure (*n* = 5). (**B**) Western blots of PPARα, CPT1a, CACT, CPT2, and β-actin in liver of the young mice, the aged mice, and the aged mice administered with AR-C17 after cold exposure. (**C**) Relative protein levels of PPARα, CPT1a, CACT, CPT2, and β-actin in liver of the young mice, the aged mice, and the aged mice administered with AR-C17 after cold exposure (*n* = 3). (**D**,**E**) The short-chain, medium-chain, and long-chain acylcarnitine (ACar) levels in plasma of the young mice, the aged mice, and the aged mice administered with AR-C17 after cold exposure (*n* = 3). Data are presented as the mean ± SEM and n indicates the number of biologically independent experiments. Statistical significance was determined using one-way ANOVA with post hoc analyses. * *p* < 0.05; ** *p* < 0.01; *** *p* < 0.001; ns, not statistically significant.

Since the liver is regarded as a hub in thermogenesis, and hepatic FA oxidation is important for BAT thermogenesis [8], we investigated the effect of AR-C17 on aging-associated hepatic FA oxidation dysfunction. The results showed that the liver mass of aged mice was increased compared with young mice, and AR-C17 reduced the liver mass of aged mice after cold exposure, with no effect on the ratio of liver weight to body weight (Figure 2A,B). Meanwhile, AR-C17 alleviated the hepatic lipid accumulation of aged mice after cold exposure, as demonstrated by the histological analysis and the hepatic TC and TG levels (Figure 2C–E). Liver-derived acylcarnitine provides a fuel source for BAT thermogenesis, and thus hepatic acylcarnitine metabolism is important for BAT thermogenesis during cold stimulation [9]. Acylcarnitine metabolism is part of lipid metabolism. Further analysis showed that AR-C17 reversed aging-associated decline in gene expressions of hepatic FA oxidation, with increased protein levels of peroxisome proliferator-activated receptor α (PPARα), CPT1a, CACT, and CPT2 in the liver of aged mice during cold stimulation (Figure 3A–C). The results indicated that the FA oxidation and acylcarnitine metabolism in the liver of aged mice were reversed by AR-C17. Moreover, we measured the acylcarnitine levels in the liver, which are a key fuel source for BAT thermogenesis during cold stimulation. The results showed that the short-chain and medium-chain acylcarnitine levels in the liver of aged mice were almost similar to those of young mice during cold stimulation, and AR-C17 just increased the C2:0, C3:0, and C12:0 of short-chain and medium-chain acylcarnitine levels in the liver of aged mice (Figure 3D). However, the long-chain acylcarnitine levels in the liver in aged mice were significantly increased by AR-C17 during cold stimulation (Figure 3E). Therefore, AR-C17 improved acylcarnitine metabolism in the liver of aged mice, reversing an aging-associated scarcity of fuel from the liver for BAT thermogenesis. Together, AR-C17 might effectively improve hepatic FA oxidation during aging.

### 3.3. AR-C17 Improved WAT Lipolysis and Increased Adipose Sirt3 Expressions during Aging

**Figure 4:**
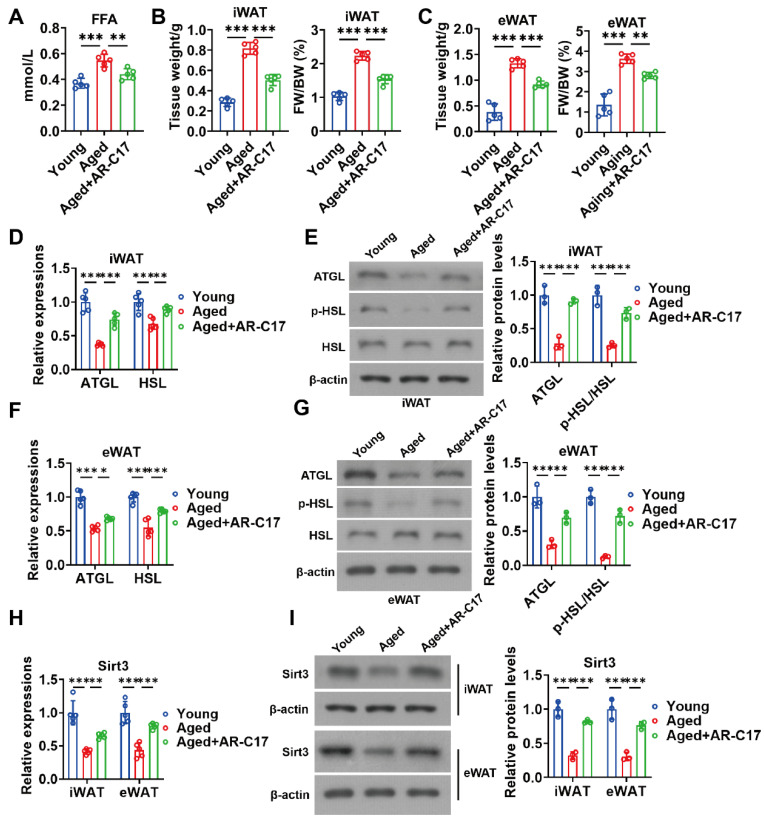
AR-C17 improved WAT lipolysis and increased adipose Sirt3 expressions during aging after cold exposure. (**A**) The serum FFA levels of the young mice, the aged mice, and the aged mice administered with AR-C17 after cold exposure (*n* = 5). (**B**) The iWAT weight and ratio of iWAT weight to body weight (FW/BW) of the young mice, the aged mice, and the aged mice administered with AR-C17 after cold exposure (*n* = 5). (**C**) The eWAT weight and ratio of eWAT weight to body weight (FW/BW) of the young mice, the aged mice, and the aged mice administered with AR-C17 after cold exposure (*n* = 5). (**D**) Relative mRNA levels of ATGL and HSL in iWAT of the young mice, the aged mice, and the aged mice administered with AR-C17 after cold exposure (*n* = 5). (**E**) Western blots of ATGL, p-HSL (Ser565), HSL, and β-actin, and relative protein levels of ATGL and p-HSL (Ser565)/HSL in iWAT of the young mice, the aged mice, and the aged mice administered with AR-C17 after cold exposure (*n* = 3). (**F**) Relative mRNA levels of ATGL and HSL in eWAT of the young mice, the aged mice, and the aged mice administered with AR-C17 after cold exposure (*n* = 5). (**G**) Western blots of ATGL, p-HSL (Ser565), HSL, and β-actin, and relative protein levels of ATGL and p-HSL (Ser565)/HSL in eWAT of the young mice, the aged mice, and the aged mice administered with AR-C17 after cold exposure (*n* = 3). (**H**) Relative mRNA levels of Sirt3 in iWAT and eWAT of the young mice, the aged mice, and the aged mice administered with AR-C17 after cold exposure (*n* = 5). (**I**) Western blots of Sirt3 and β-actin, and relative protein levels of Sirt3 in iWAT and eWAT of the young mice, the aged mice, and the aged mice administered with AR-C17 after cold exposure (*n* = 3). Data are presented as the mean ± SEM and n indicates the number of biologically independent experiments. Statistical significance was determined using one-way ANOVA with post hoc analyses. * *p* < 0.05; ** *p* < 0.01; *** *p* < 0.001.

During cold stimulation, WAT lipolysis promotes hepatic acylcarnitine production [9]. Therefore, WAT lipolysis may be important for hepatic lipid metabolism. To investigate the effect of AR-C17 on the WAT lipolysis of aged mice, we measured the serum levels of FFA produced by lipolysis and the WAT lipolysis-associated gene and protein expressions. The result showed that AR-C17 reduced the high serum level of FFA in aged mice during cold stimulation (Figure 4A), indicating that AR-C17 might promote the clearance of serum FFA in aged mice. Further analysis showed that the mass of iWAT and eWAT in aged mice during cold stimulation was reduced by AR-C17, with elevated expressions of lipolysis-associated genes and proteins (Figure 4B–G). The results suggested that an aging-associated impairment of WAT lipolysis was improved by AR-C17 during cold stimulation, thus promoting hepatic acylcarnitine production. In addition, we have reported that AR-C17 improves adipose metabolism via regulating Sirt3 [22]. Then, we measured the gene and protein expressions of Sirt3 in iWAT and eWAT. The results showed that the gene and protein expressions of Sirt3 in iWAT and eWAT of aged mice were downregulated compared with young mice after cold exposure, and AR-C17 reversed the aging-associated downregulation of Sirt3 gene and protein expressions in WAT (Figure 4H,I). Therefore, we speculated that WAT Sirt3 might be important for WAT lipolysis and subsequent hepatic FA oxidation, and AR-C17 might influence WAT lipolysis and hepatic FA oxidation by regulating the WAT Sirt3 of mice during cold stimulation.

### 3.4. AR-C17 Failed to Reinforce WAT Lipolysis during Aging in the Absence of Adipose Sirt3

**Figure 5:**
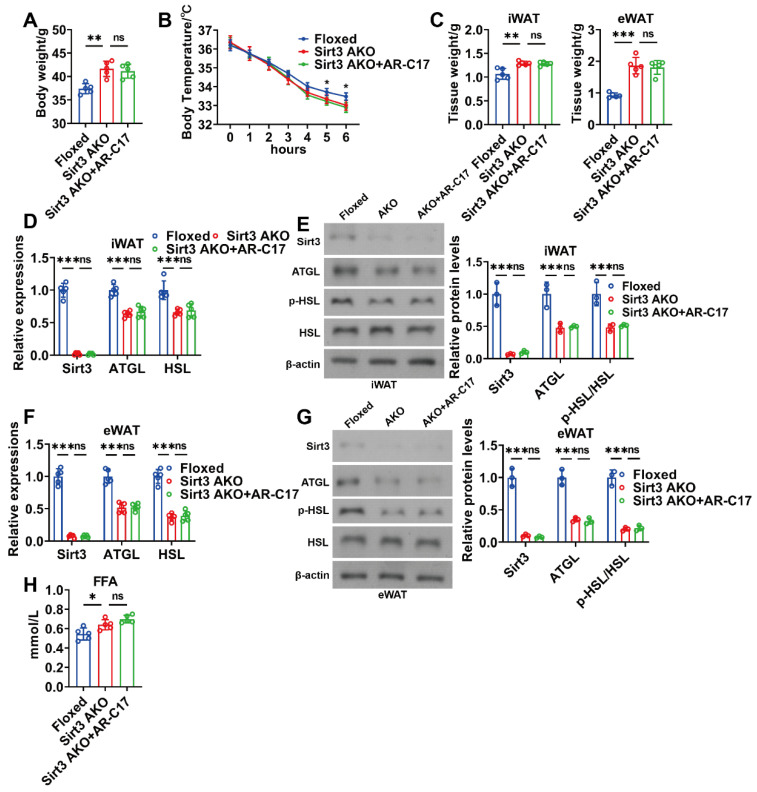
AR-C17 failed to reinforce WAT lipolysis during aging in the absence of adipose Sirt3 after cold exposure. (**A**) Body weight of the Floxed mice, the Sirt3 AKO mice, and the Sirt3 AKO mice administered with AR-C17 (*n* = 5). (**B**) Core body temperature of the Floxed mice, the Sirt3 AKO mice, and the Sirt3 AKO mice administered with AR-C17 during cold exposure (*n* = 5). (**C**) The iWAT and eWAT weight of the Floxed mice, the Sirt3 AKO mice, and the Sirt3 AKO mice administered with AR-C17 after cold exposure (*n* = 5). (**D**) Relative mRNA levels of Sirt3, ATGL, and HSL in iWAT of the Floxed mice, the Sirt3 AKO mice, and the Sirt3 AKO mice administered with AR-C17 after cold exposure (*n* = 5). (**E**) Western blots of Sirt3, ATGL, p-HSL (Ser565), HSL, and β-actin, and relative protein levels of Sirt3, ATGL, and p-HSL (Ser565)/HSL in iWAT of the Floxed mice, the Sirt3 AKO mice, and the Sirt3 AKO mice administered with AR-C17 after cold exposure (*n* = 3). (**F**) Relative mRNA levels of Sirt3, ATGL, and HSL in eWAT of the Floxed mice, the Sirt3 AKO mice, and the Sirt3 AKO mice administered with AR-C17 after cold exposure (*n* = 5). (**G**) Western blots of Sirt3, ATGL, p-HSL (Ser565), HSL, and β-actin, and relative protein levels of Sirt3, ATGL, and p-HSL (Ser565)/HSL in eWAT of the Floxed mice, the Sirt3 AKO mice, and the Sirt3 AKO mice administered with AR-C17 after cold exposure (*n* = 3). (**H**) The serum FFA levels of the Floxed mice, the Sirt3 AKO mice, and the Sirt3 AKO mice administered with AR-C17 after cold exposure (*n* = 5). Data are presented as the mean ± SEM and n indicates the number of biologically independent experiments. Statistical significance was determined using one-way ANOVA with post hoc analyses. * *p* < 0.05; ** *p* < 0.01; *** *p* < 0.001; ns, not statistically significant.

AR-C17 can be found in plasma and adipose tissue biopsies of the human body [27], while whether AR-C17 can enter the liver remains unclear. Therefore, it could not be concluded that AR-C17 directly regulated hepatic FA oxidation. Since hepatic FA oxidation can be promoted by WAT lipolysis, we speculated that AR-C17 might improve the hepatic FA oxidation of aged mice via regulating WAT lipolysis, and Sirt3 might be the key regulator in AR-C17-regulated WAT lipolysis. To investigate whether Sirt3 underlies the WAT lipolysis of aged mice regulated by AR-C17, adipose-specific Sirt3 knockout (Sirt3 AKO) mice were established. The result showed that Sirt3 AKO mice displayed a higher body weight than that of the Floxed mice during aging, and AR-C17 failed to reduce the body weight of aging Sirt3 AKO mice (Figure 5A). The knockout of Sirt3 in adipose reduced the body temperature of mice during cold stimulation, and AR-C17 was unable to reverse the cold-sensitive phenotype of aging Sirt3 AKO mice (Figure 5B). Furthermore, the knockout of Sirt3 in adipose increased the weight of both iWAT and eWAT of aged mice, which was unable to be reduced by AR-C17 during cold stimulation (Figure 5C). Meanwhile, the lipolysis-associated gene and protein expressions in iWAT and eWAT of aged mice were downregulated in the absence of adipose Sirt3, and AR-C17 failed to upregulate the lipolysis-associated gene and protein expressions in the iWAT and eWAT of aging Sirt3 AKO mice during cold stimulation (Figure 5D–G). The results indicate that AR-C17 failed to reverse the aging-associated impairment of WAT lipolysis in the absence of adipose Sirt3. In addition, the knockout of Sirt3 in adipose increased the serum FFA level of aged mice during cold stimulation, which was unimproved by AR-C17 (Figure 5H). Therefore, adipose Sirt3 is important for the improvement of AR-C17 in the aging-associated impairment of WAT lipolysis.

### 3.5. AR-C17 Failed to Improve Hepatic Fatty Acid Oxidation during Aging in the Absence of Adipose Sirt3

**Figure 6:**
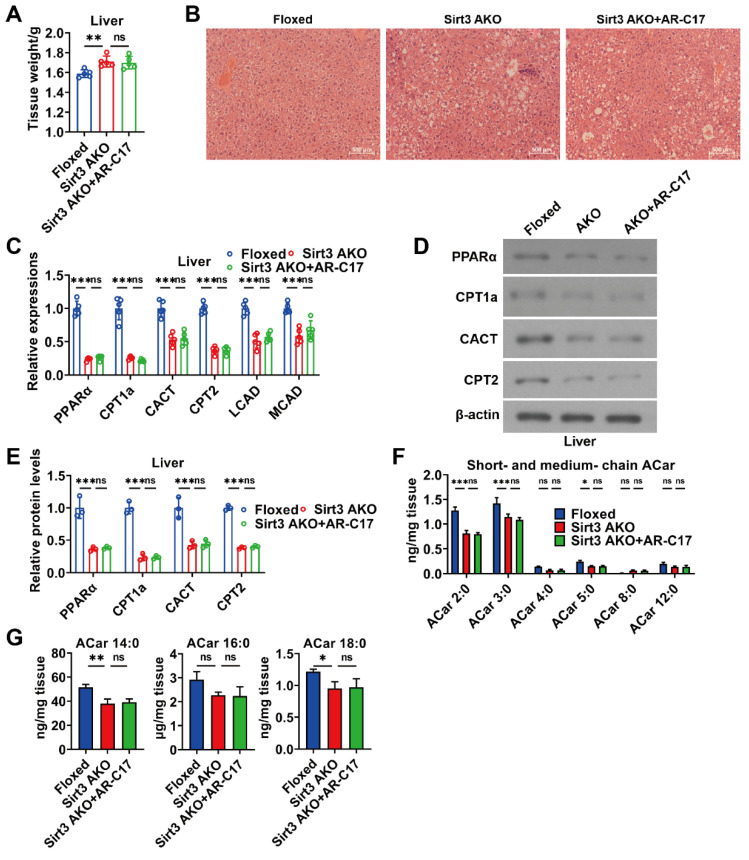
AR-C17 failed to improve hepatic FA oxidation during aging in the absence of adipose Sirt3 after cold exposure. (**A**) The liver weight of the Floxed mice, the Sirt3 AKO mice, and the Sirt3 AKO mice administered with AR-C17 after cold exposure (*n* = 5). (**B**) Representative H&E staining of liver from the Floxed mice, the Sirt3 AKO mice, and the Sirt3 AKO mice administered with AR-C17 after cold exposure. (**C**) Relative mRNA levels of PPARα, CPT1a, CACT, CPT2, LCAD, and MCAD in the liver of the Floxed mice, the Sirt3 AKO mice, and the Sirt3 AKO mice administered with AR-C17 after cold exposure (*n* = 5). (**D**) Western blots of PPARα, CPT1a, CACT, CPT2, and β-actin in the liver of the Floxed mice, the Sirt3 AKO mice, and the Sirt3 AKO mice administered with AR-C17 after cold exposure. (**E**) Relative protein levels of PPARα, CPT1a, CACT, and CPT2 in the liver of the Floxed mice, the Sirt3 AKO mice, and the Sirt3 AKO mice administered with AR-C17 after cold exposure (*n* = 3). (**F**,**G**) The short-chain, medium-chain, and long-chain acylcarnitine (ACar) levels in the plasma of the Floxed mice, the Sirt3 AKO mice, and the Sirt3 AKO mice administered with AR-C17 after cold exposure (*n* = 3). Data are presented as the mean ± SEM and n indicates the number of biologically independent experiments. Statistical significance was determined using one-way ANOVA with post hoc analyses. * *p* < 0.05; ** *p* < 0.01; *** *p* < 0.001; ns, not statistically significant.

Supposing that AR-C17 directly regulates hepatic FA oxidation during aging, AR-C17 can improve aging-associated hepatic FA oxidation dysfunction independent of adipose. We analyzed the hepatic FA oxidation of aged mice in the absence of adipose Sirt3. The results showed that the knockout of Sirt3 in adipose increased the liver weight and promoted the hepatic lipid accumulation of aged mice, while AR-C17 had no effect on liver weight and the hepatic lipid accumulation of aging Sirt3 AKO mice during cold stimulation (Figure 6A,B). Further analysis showed that the knockout of Sirt3 in adipose downregulated the gene expressions of hepatic FA oxidation and the protein levels of PPARα, CPT1a, CACT, and CPT2 in the liver of aged mice during cold stimulation, which were not significantly increased by AR-C17 (Figure 6C–E). The results indicated that the FA oxidation and acylcarnitine metabolism in the liver of aged mice could not be reversed by AR-C17 in the absence of adipose Sirt3. Moreover, we measured the acylcarnitine levels in the liver of aged mice in the absence of adipose Sirt3. The results showed that the knockout of Sirt3 in adipose reduced the short-chain and long-chain acylcarnitine levels in the liver of aged mice during cold stimulation without the changes in medium-chain acylcarnitine levels, while the hepatic acylcarnitine levels of aged mice could not be increased by AR-C17 in the absence of adipose Sirt3 (Figure 6F,G). Therefore, AR-C17 failed to reverse the aging-associated scarcity of fuel from the liver for BAT thermogenesis in the absence of adipose Sirt3. In conclusion, AR-C17 could not directly regulate hepatic FA oxidation during aging, and adipose Sirt3 is indispensable for AR-C17 to improve the aging-associated hepatic FA oxidation dysfunction of aged mice in response to cold.

**Figure 7:**
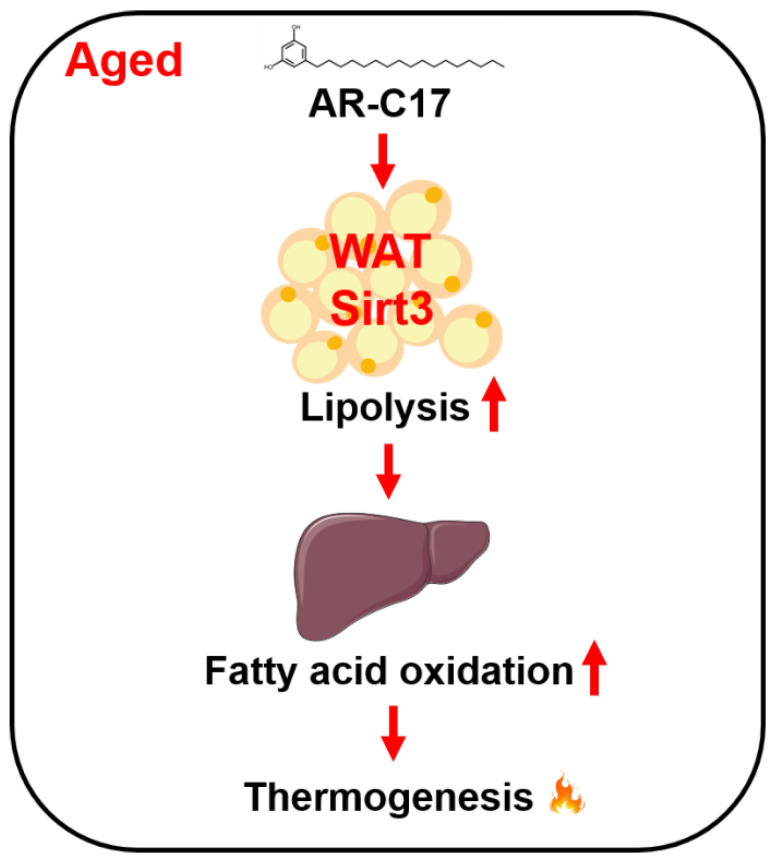
Schematic diagram of the working model in this study.

Altogether, our study revealed that AR-C17 might improve aging-associated hepatic FA oxidation dysfunction to enhance adaptive thermogenesis via regulating adipose Sirt3 (Figure 7).

## 4. Discussion

AR-C17 has been reported to regulate various metabolic processes in different tissues and microenvironments by targeting the Sirt3 signaling pathway. AR-C17 can attenuate oxidative damage and apoptosis through regulating the Sirt3 signaling pathway in neurocytes [19]. AR-C17 has been demonstrated to protect against the mitochondrial dysfunction of adipocytes through regulating Sirt3-mediated autophagy [16]. AR-C17 can protect against arteriosclerosis in endothelial cells and apolipoprotein E-deficient mice by modulating Sirt3 signaling [21]. Furthermore, AR-C17 can reduce neuroinflammation by activating Sirt3 signaling [18]. AR-C17 has also been demonstrated to ameliorate obesity-associated skeletal muscle mitochondrial dysfunction through Sirt3-mediated mitophagy [20]. In our previous studies, we demonstrated that AR-C17 could modulate the adipose thermogenesis of aged mice via regulating the Sirt3-AMP (adenosine monophosphate)-activated protein kinase pathway and enhancing the acylcarnitine metabolism of BAT [14,22]. However, there is no evidence that AR-C17 could regulate hepatic function or mediate organ crosstalk. In the present study, we demonstrated that AR-C17 might improve the hepatic FA oxidation of aged mice via regulating adipose Sirt3, which is important for WAT lipolysis. Knockout Sirt3 in the adipose of aged mice eliminated the improvement in hepatic FA oxidation by AR-C17. The results indicated that AR-C17 was unable to directly regulate hepatic FA oxidation, and that adipose Sirt3 is crucial to AR-C17-mediated organ crosstalk between WAT and the liver.

Sirt3, a NAD^+^-dependent mitochondrial deacetylase, plays a critical role in the regulation of metabolic homeostasis [24]. Many bioactive ingredients from food have been demonstrated to regulate Sirt3 in vivo and in vitro. Sesamin and sesamol can attenuate oxidative stress via regulating the Sirt3 signaling pathway in human neuronal cells [29]. The cardioprotective effects of resveratrol are exerted through the regulation of Sirt3 [30]. Pine nut antioxidant peptides have been demonstrated to ameliorate the memory impairment of mice via regulating Sirt3-induced synaptic plasticity [31]. Chinese traditional vinegar-powder-derived melanoidins inhibit inflammation and oxidative stress in macrophages via activating Sirt3 [32]. Dihydromyricetin has been proven to attenuate oxidative stress and promote autophagy via regulating Sirt3 signaling in hepatocytes [33]. Moreover, highland barley tea polyphenols have been demonstrated to alleviate skeletal muscle fibrosis and improve the mitochondrial dysfunction of myocytes via regulating the Sirt3 signaling pathway [34,35]. However, there is no evidence that the bioactive ingredients from food can mediate organ crosstalk via regulating Sirt3 to modulate systemic metabolic homeostasis. Our study suggests that AR-C17 mediates organ crosstalk between WAT and the liver, improving hepatic FA oxidation via regulating Sirt3 in WAT.

WAT lipolysis promotes hepatic acylcarnitine production during cold stimulation, leading to an increase in plasma acylcarnitines, which are required for adaptive thermogenesis by BAT [9]. Liver is the hub of the WAT–liver–BAT crosstalk that mediates thermogenesis connected by acylcarnitine [8]. In this study, AR-C17 mediated organ crosstalk between WAT and the liver via regulating Sirt3 in WAT. Since we have previously revealed that AR-C17 directly regulates the BAT thermogenesis of aged mice via targeting Sirt3, AR-C17 might also mediate hepatic FA oxidation through regulating Sirt3 in BAT. Many pieces of evidence suggest that BAT, as a secretory organ, can regulate hepatic functions through various BAT-derived secretory factors [36]. BAT secretes neuregulin 4 to regulate metabolic homeostasis by inhibiting hepatic lipogenesis [37]. The BAT-derived phospholipid transfer protein is a mediator of BAT–liver interorgan communication, contributing to the regulation of systemic glucose and lipid metabolic homeostasis [38]. Furthermore, exclusively BAT-expressed UCP1 mediates the clearance of liver extracellular succinate, regulating hepatic inflammatory [39]. BAT-derived maresin 2 has been proven to partially reduce inflammation in obesity by targeting macrophages in the liver [40]. Most recently, BAT-derived extracellular vesicle-miR-378a-3p has been demonstrated to regulate hepatic gluconeogenesis [41]. Therefore, BAT regulates hepatic metabolism through its various secretory factors. In our study, we cannot exclude the possibility that AR-C17 can mediate hepatic FA oxidation through regulating secretory factors in BAT, which is the limit of our study and should be investigated in future.

## 5. Conclusions

In this study, AR-C17 improves aging-associated hepatic FA oxidation dysfunction through enhancing WAT lipolysis via the regulation of adipose Sirt3. Sirt3 plays an important role in the regulation of systemic metabolic homeostasis by AR-C17. This study enriches the metabolic regulation function of AR-C17 and expands the application of WG food in the field of anti-aging.

## Data Availability

The data presented in this study are available upon request from the corresponding author due to privacy.

## Supplementary Materials

The following supporting information can be downloaded at: https://www.mdpi.com/article/10.3390/nu16070978/s1, Table S1: Primer sequences.
